# Active Methodologies in Higher Education: Perception and Opinion as Evaluated by Professors and Their Students in the Teaching-Learning Process

**DOI:** 10.3389/fpsyg.2020.01703

**Published:** 2020-08-04

**Authors:** Emilio Crisol-Moya, María Asunción Romero-López, María Jesús Caurcel-Cara

**Affiliations:** ^1^Department of Pedagogy and School Organization, University of Granada, Granada, Spain; ^2^Department of Pedagogy and School Organization, University of Granada, Granada, Spain; ^3^Department of Physical and Education, University of Granada, Granada, Spain

**Keywords:** active methodology, higher education, professor, student, perception, opinion, learning process

## Abstract

The goal of this study is both to determine the opinion that professors and students at the university have of active methodologies and to describe the perception and opinion of the modes of organization, methodological focuses, and evaluation systems that define the teaching-learning process. On surveying the professors and the students in their classes, we found significant differences in 32 of the 92 variables in common. The content of these results shows that professors and students are believe they are making progress toward a learning-centered model, that implementation of active methodologies implies new functions in their teaching practice.

## Introduction

Teaching and learning practices in higher education are undergoing a number of changes that have significant implications for the nature of students’ learning experience. The traditional approach to teaching in Spain, as in many parts of the world, involved one-way transmission from lecturer to students (Ituma, 2011).

From various studies analyzed (García Valcárcel, 1993; Alonso and Méndez, 1999; Kember and Kwan, 2000; Biggs, 2005; Monereo and Pozo, 2006; Kember, 2009; Attard et al., 2010; Hynes, 2017; Gómez and Gil, 2018; Cabral and Duarte, 2019; Dominguez et al., 2019; Zamora-Polo and Sánchez-Martín, 2019), we confirm continued use of the traditional model – also called the knowledge transmission or teacher-centered model, which focuses on the teacher, transmission of information, and expository style, but also a greater presence of the model that focuses on student learning, also called learning facilitation, the constructivist model, student-centered learning model or learning paradigm (Kolb, 1976; Imbernón and Medina, 2006; León and Crisol, 2011). The two orientations coexist in teaching methodology, understood here as different modes of organization, methodological focus, and evaluations system that stress the reproduction of knowledge and the role of methodology in the construction and/or transformation of knowledge (Samuelowicz and Bain, 2002). In higher education, calls have been made for active learning experiences that place the student at the center of learning rather than accepting students as passive listeners (Boyer, 1990; Felder and Brent, 1996; Qualters, 2001; Jungst et al., 2003; Machemer and Crawford, 2007; Zamora-Polo and Sánchez-Martín, 2019).

## From the Traditional Teaching Model to Student-Centered Learning

The teaching-centered model gives special importance to the figure of the teacher, who is considered as the fundamental source of information and knowledge. In this model, the teacher is the one who knows, and it is his/her responsibility to transmit that knowledge well, leaving students the sole task of reproducing the knowledge (Gargallo-López et al., 2017). Further, responsibility for curricular design and development belongs exclusively to the teacher, including mode of organization of the instruction, choice of content, and teaching methods and evaluation procedures. The same is true for transformation of knowledge. In this case, reproduction is sought as a product of learning. This model does not seek student involvement in either construction of knowledge or decision-making about how that knowledge about students’ learning; it does not stress development of skills like cooperative work. It focuses on competence rather than cooperation, with minimal and one-way interaction between student and teacher. Instruction will only occasionally be two-way in order to keep students’ attention or ensure understanding of the content treated in order to resolve questions. Ordinarily, such classes are based on explanation, using lecture, student note-taking, and memorization so that students can repeat the knowledge later. Students are usually evaluated by the traditional exam. The learning-centered model, in contrast, stresses the student’s learning. Knowledge is understood as personal construction, fruit of cooperation between teacher and students. The product of learning should be exchange of knowledge. Although the teacher is responsible for curricular design, this model requires joint work of the teacher and his/her colleagues, as well as cooperation with the students. The student is invited to design his/her learning pathways and to commit him- or herself actively in the process (Machemer and Crawford, 2007), such that the responsibility for organization and transformation of knowledge is shared. The student’s conceptions are used as the basis for preventing errors and promoting conceptual change. Teacher-student interaction is two-way to negotiate meanings. The student’s cooperative work is promoted for joint construction of knowledge and development of skills, attitudes and values necessary in his/her student and subsequent professional life. This method seeks a significant evaluation methodology that uses diverse sources of information gathering and that gives the students feedback (Hernández, 2012), helping them to mobilize processes of self-evaluation (Hannafin, 2012) and self-regulation of the learning process.

The literature shows quite a few publications recommending the learning-centered model in various areas of knowledge (Tagg, 2003; Zabalza, 2006, 2011; Menachery et al., 2008; Prieto and coord, 2008; McLean and Gibbs, 2010; Bista, 2011; Brackin, 2012; Mostrom and Blumberg, 2012; Campbell, 2012; Hunt and Chalmers, 2013; Nitza, 2013; Sue, 2014; Cebrián-de-la-Serna et al., 2015; Schweisfurth, 2015), as well as studies analyzing experiences implemented (Tien et al., 2002; Koles et al., 2005; Tessier, 2007; Armbruster et al., 2009; Salaburu et al., 2011; Roy and McMahon, 2012; Bruehl et al., 2014; Chen et al., 2015; Lucieer et al., 2016; Zamora-Polo and Sánchez-Martín, 2019). This body of research indicates that we are undergoing a methodological renewal that involves the use of new forms of organization (modes of organization), teaching methodologies (methodological focuses), and evaluative processes (evaluation systems) in accordance with new professional profiles and a new way of understanding learning that is crucial for the transition from a teaching-centered methodology to a one centered in learning that encourages active learning.

Of the many broad definitions of active learning, all basically involve something more than passive listening (Qualters, 2001; Lammers and Murphy, 2002; Jungst et al., 2003). Active learning is a broad, commonly used term “generally defined as any instructional method that engages students in the learning process” (Prince, 2004, p. 223). The student’s active participation requires the implementation of active methodologies with repercussions for both the educational process and the mechanisms used to evaluate the degree and quality of learning acquired. Thus, lectures have lost their leading role as the only or main method in university classrooms and must be combined with other methodologies, termed active: seminars, learning projects, mentored projects, readings, reviews, document analyses, case studies, bibliographic searches, problem-based learning, virtual platforms, practical class sessions, etc. – all more oriented to the student’s independent work and active learning. Active learning does not negate the need for lectures, but it provides opportunities for students to reflect, evaluate, analyze, synthesize, and communicate on or about the information presented (Fink, 2003).

The presence of active methods in university classrooms will be effective as long as the professor takes the student’s participation into account in organizing and proposing the teaching and learning methodologies, as well as the evaluation methods. Although many experiences of the implementation of active methodologies have been observed (Fernández-Pérez, 1989; Exley and Dennick, 2007; López-Noguero, 2007; Rué, 2007; López-Pastor et al., 2011) in various subjects in different fields of knowledge, many quite innovative teachers – whether employing active methodological strategies or not – continue to base their classes on dictation, readings, explanations, etc. that leave the student in a state of passivity, preventing students from achieving significant learning. Educators have proposed widely varying examples of pedagogical strategies or techniques for active learning including, for instance, case studies, team projects, simulations and role playing, internships, peer tutoring, and challenging discussions (Chickering and Gamson, 1987; Bonwell and Eison, 1991; Meyers and Jones, 1993; Chi, 2009; Carr et al., 2015). What do these teaching strategies have in common? The theory behind these techniques is based on a constructivist view of learning. Constructivism posits that people build knowledge by acting on the world around them and reflecting on their experiences. Being constructive means ensuring that all components of the teaching-learning process are developed unanimously, so that both the methodological focuses (teaching methods) and the evaluation systems (evaluation procedures) are designed to achieve the desired competences and learning outcomes (Gibbs, 1994; Biggs, 2005).

But the problem stems from the fact that this methodology, which fosters active learning, is often misapplied or not applied at all, meaning that active methodologies are present only in theory. It is not enough that the use of active methodologies attributes a very significant role to the student, who constructs his/her knowledge from certain guidelines, activities, or scenarios designed by the professor. Through these activities, the professor must encourage the student to (Crisol, 2013): become responsible for his/her own learning, developing skills in searching for, selecting, analyzing, and evaluating information, participate in activities that enable him/her to exchange experiences and opinions with peers, commit him- or herself in processes of reflection on what to do, how to do it, and what results to achieve, proposing specific actions in order to improve, interact with his/her environment to intervene socially and professionally in it, through activities such as projects, case studies, and problem solving, develop autonomy, critical thinking, collaborative attitudes, professional skills, and self-evaluation capability.

These key issues help to determine how to organize students’ learning, how to evaluate students, and how both professor and student should act. Since these issues represent the three fundamental components of these methodologies, they formed the major focus of the study we present.

First, we have the organizational component, that is, the scenario or scenarios in which the teaching-learning processes will be developed. In this study, these are determined as modes of organization, following the classification by De Miguel (2006), De Miguel and coord. (2009) and the Ministry of Education and Culture [MEC] (2006): theoretical classes, seminars, workshops, practical classes, tutorials, outside internships, independent individual work and study, and group work.

The second part forms the procedural technical component, formulated as methodological focuses, also following the classification presented by De Miguel (2006), De Miguel and coord. (2009): participatory lecture, oral presentation of student projects, seminar, case studies, problem-based learning, portfolios, independent work, cooperative work, project-oriented learning, learning contracts, and concept maps.

The last component is the evaluative, determined as evaluation systems: objective tests, long-answer tests, oral exams, papers and projects, reports/memoranda on practical class sessions, tests on execution of real tasks, self-evaluation systems, observation techniques, portfolios, and concept maps.

It is not easy to shift from a teaching-centered focus to one centered on learning (Heise and Himes, 2010). This shift requires organizational changes, new infrastructures and equipment, cooperative work by professors, and integrated curriculum design (De La Sablonnière et al., 2009), all of which require motivation and commitment from professors and students, as well as training programs for professors (Maclellan, 2008), since they continue to organize and plan around lecture classes.

De La Sablonnière et al. (2009) have, however, performed studies on students’ perceptions of a variety of class environments. Since the research on students’ perceptions of active learning opportunities and environments is limited and contradictory (Machemer and Crawford, 2007), this article provides data from a study whose fundamental goal was to determine the opinion that professors and students at the University of Granada (UGR) have of active methodologies and to describe the perception (frequency of use) and opinion (appropriateness of use) of the modes of organization, methodological focuses, and evaluation systems that define the teaching-learning process. This study is based on the conviction that there is a need for empirical data to help to improve the quality of teaching-learning in the university.

## Materials and Methods

### Participants

The study was performed at the UGR, whose teaching centers are divided into five areas of knowledge: Arts and Humanities, Sciences, Health Sciences, Social Sciences and Law, and Technical Sciences (Engineering and Architecture). The population in this study is the set of all professors and students of the UGR.

According to the UGR’s Research Faculty Services (Personal Docente Investigador), during the 2016/2017 academic year the faculty was composed of a total of 4126 professors (54.7% men and 45.3% women) affiliated with the different areas. The data on students published by the Office for Data, Information, and Planning gives the student population as 46 483 (60.40% men and 39.60% women).

This study used a non-probabilistic convenience sample according to the students and teachers that could be accessed. This sampling method ensures that the sample represents the various subgroups of a population based on the characteristics of the population in the exact proportion the researcher wishes (Hernández et al., 2006). From the total population of professors at the UGR, 32 professors participated in this study, along with the students in their respective classes.

By sex, the faculty were distributed as follows: 34% (*n* = 11) were men and 66% women (*n* = 21). As to age, 37.5% (6 men and 6 women) were 41–50 years of age, 18% (1 man and 6 women) 51–60, 25% (2 men and 6 women) 31–40, and 18.8% (2 men and 4 women) under 30.

As to discipline, 46.9% (15 professors) of the faculty who participated in the study belonged to the field of Social Science and Law, 25% (8 professors) to Arts and Humanities, 12.5% (4 professors) to Technical Sciences, 9.4% (3 professors) to Health Sciences, and 6.3% (2 professors) to Sciences.

As to education, 65.6% of the professors said that they had received specific training in active methodologies. Although a total of 84.4% used active methodologies in their teaching, only 59.4% (19 professors) took their students’ opinion into account when proposing the teaching-learning methodologies.

As to the students, we had 32 class sections (one class per instructor), comprising a total of 1234 students. Of this total, 54.7% (675 students) were women and the remaining 45.3% (559 students) men. By age, 77.3% (*N* = 954 students) were 18–22 years old, 22.6% (*N* = 279 students) 23–27, and only 10% (*N* = 1 student) over 28.

By field of knowledge, 39.2% (*N* = 484 students) belonged to Social Sciences and Law, 18.3% (*N* = 226 students) to Arts and Humanities, 17.3% (*N* = 214 students) to Health Sciences, and 1.1% (*N* = 14 students) to Sciences. 18.8% (*N* = 233 students) percent of all students are Physical Education students.

Regarding year in degree program, 40.8% (*N* = 503 students) were registered in the first year of their degree programs, 27.5% (*N* = 339 students) in their second year, 15.6% (*N* = 192 students) in their third year, and 16.2% (*N* = 200 students) in their fourth year.

Of the total student population, 67.1% (*N* = 828 students) stated that their professors used active methodologies in teaching, while 32.9% (*N* = 406 students) believed that their professors used traditional methodologies. Only 30% (*N* = 369 students) of those who believed their professors used active methodologies believed that this method took the students’ opinion into account when proposing the methodologies to be used in class; 37.2% (*N* = 459 students) of the students believed that the professors did not take their opinion into account in the methodological approach to the teaching-learning process. In contrast, 27.2% (N = 336 students) of the students believed that the professors neither used active methodologies nor took students’ opinions into account in establishing the methodologies. The other 5.6% (*N* = 70 students) believed that the professor took students’ opinion in determining the methodology in the teaching-learning process even if he/she did not use active methodologies.

### Design of the Study and Instruments

The research presented here is quantitative, and transversal and descriptive in approach. Starting from an exploratory, descriptive, and comparative research model, it explores the opinion that university professors and students have of the use of active methodologies at the UGR. It describes the perception of these two groups (professors and students) of the different modes of organization, methodological focuses, and evaluation systems, and compares the opinions and perceptions of the professors with those of the students.

This study is developed within the framework of an analytic-synthetic method, starting from use of the questionnaire as research instrument in order to approximate reality in an objective and generalizable way.

The goal of this study is both to determine the opinion that professors and students at the UGR have of active methodologies and to describe the perception (frequency of use) and opinion (appropriateness of use) of the modes of organization, methodological focuses, and evaluation systems that define the teaching-learning process, based on the following declarative hypotheses:

H1: There are statistically significant differences between the professors’ opinions and those of the students in their classes concerning the use of active methodologies.
H2: There are statistically significant differences between the perception (frequency of use) and opinion (level of appropriateness) of the professors and of the students in their classes concerning the use of modes of organization, methodological focuses, and evaluation systems.

For this study, we chose the research instrument of a “survey” questionnaire (Buendía and Colas, 1997), understood as a set of carefully prepared questions on the actions and issues considered relevant to the research and to be verified by the population or sample participating in the study (Sierra Bravo, 1988). In other words, the goal of this instrument is to obtain information on the study population’s relation to the study variables (professors, students, fields of knowledge) in a systematic and orderly way.

The questionnaires used “Opinion and Perception of the professors concerning the use of active methodologies at the University of Granada (OPPUMAUGR),” and “Opinion and Perception of the students concerning the use of active methodologies at the University of Granada (OPEUMAUGR)” (León and Crisol, 2011; Crisol, 2013). The questionnaires have not been published previously and were developed from the bibliography and the researchers’ relationships to the topic of study (Johnson et al., 1999; Marín and Teruel, 2004; De Miguel, 2006, De Miguel and coord., 2009; Ministry of Education and Culture [MEC], 2006; Monereo and Pozo, 2006; Barkley et al., 2007; Moust et al., 2007; Imbernón and Medina, 2008; Sánchez, 2008; Caurcel et al., 2009; Fernández, 2009; Learreta et al., 2009; Vallejo-Ruiz and Molina-Saorín, 2011).

Both were distributed in two parts, the first on Opinion of the use of active methodologies, and the second on Perception and Opinion of the teaching-learning process.

The OPPUMAUGR questionnaire is composed of 126 items. The first part “Opinion of active methodologies,” has 68 items divided into 5 dimensions: methodological renewal (13 items), use of active methodologies (28 items), teaching professional context (9 items), context in the university (6 items), and context in university classrooms (10 items). The second part, “Perception and Opinion of the teaching-learning process,” is composed of 60 items divided into 3 dimensions: modes of organization (16 items), methodological focuses (22 items), and evaluation systems (22 items).

The OPEUMAUGR questionnaire, in contrast, is composed of 93 items. The first part “Opinion of active methodologies,” has 32 items in 4 dimensions: methodological renewal (11 items), use of active methodologies (9 items), context in the university (4 items), and context in university classrooms (8 items). The second part has the same structure as the OPPUMAUGR questionnaire.

The first part of the questionnaires uses a Likert-type scale with 4 degrees of response, 1: Disagree completely, 2: Disagree, 3: Agree, and 4: Agree completely. The second part, for the “Frequency of use (perception)” uses the following degrees of response 1: Not at all, 2: A little, 3: Some 4: A lot. For “Appropriateness of use (opinion),” the responses are 1: Completely inappropriate, 2: Not very appropriate, 3: Appropriate, and 4: Very appropriate.

Both questionnaires have a high coefficient of reliability. The OPPUMAUGR coefficient is.893, with a confidence level of 95% (*p* ≤ 0.05). The OPEUMAUGR coefficient is 0.933, at 95% (*p* ≤ 0.05) confidence level.

### Procedure and Date Analysis

To gather data on the professors, we used the “Limesurvey” online tool for survey administration, which enabled us to translate the data directly to the SPSS tool, as well as to send multiple emails, permit participants to save the scale without completing it fully so that they could return later to complete it, and remind the participants to complete and submit their response to the scale.

Data collection for the groups of students was performed face to face, explaining to each class goal of the study and the subsequent use of the data obtained.

For the analysis, we used descriptive statistics and differential analysis using the T-Student for related samples. This method enabled us to determine the statistically significant differences between the 92 items in common between the faculty and their classes of students.

## Results and Discussion

On surveying the professors and the students in their classes, we found significant differences in 32 of the 92 variables in common. To facilitate interpretation of the results, we present them in two sections: differences between the opinion of the professors and the students in their classes on active methodologies, and differences between the perception and opinion of the professors and the students in their classes on the teaching-learning process (modes of organization, methodological focuses, and evaluation systems).

We now present the descriptive analysis and comparison of means for each item within its dimension. The tables present the scores (mean, *t*-test, and two-tailed Sig.) of the items for which we found significant differences.

### Differences Between the Professor’s Opinion and Those of Their Students on Active Methodologies

We obtained statistically significant differences in a total of 12 items of the dimensions evaluated by both professors and students concerning the use of active methodologies.

By dimension, for the dimension “methodological renewal,” Table 1 confirms that the professors show greater agreement with their students in observing that the professor’s work style is different when he/she uses active methodologies in the classroom; that the use of new teaching methodologies is accompanied by new models of evaluation; that the professor uses different pedagogical methods depending on the characteristics of the class; that the lectures are usually accompanied by other modes of instruction; and that the lecture is accompanied increasingly by active methodologies (Table 1).

**TABLE 1:** T-Student for related samples.

Items	Mean teachers	Mean students	*t*	*P*
The professor’s work style is different when he/she uses active methodologies in the classroom.	3.69	2.68	6.082	0.000
The use of new teaching methodologies is accompanied by new models of evaluation.	3.56	2.72	3.134	0.004
Different pedagogical methods are used depending on the students’ characteristics.	3.19	2.15	5.188	0.000
Lectures are usually accompanied by other modes of teaching.	3.28	2.51	4.412	0.000
Lecturing is increasingly accompanied by active methodologies.	3.25	2.71	4.295	0.000

*Methodological renewal.*

In the dimension “use of active methodologies,” Table 2 shows, overall, that the professors again agree more than their students in evaluating the following statements: the use of active methodologies fosters interdisciplinarity of content; active methodologies promote the acquisition of tools for independent learning; use of active methodologies fosters research in the classroom; and use of active methodologies fosters group work and learning among students.

**TABLE 2:** T-Student for related samples.

Item	Mean teachers	Mean students	*t*	*P*
The use of active methodologies fosters interdisciplinarity of content.	3.19	2.68	2.739	0.022
Active methodologies promote the acquisition of autonomous learning tools.	3.31	2.79	2.895	0.007
The use of active methodologies fosters research in the classroom.	3.25	2.61	3.575	0.001
The use of active methodologies fosters group work and learning among students.	3.25	2.85	2.420	0.010

*Use of active methodologies.*

As to “context in the university,” Table 3 shows, again, that the professors agree and the students disagree that the spaces dedicated to teaching do not facilitate the use of active methodologies and that infrastructure and equipment are designed for lectures. Although we find statistically significant differences between professors’ and students’ opinion that the high number of students per class hinders the use of active methodologies, both professors and students agree in this case.

**TABLE 3:** T-Student for related samples.

Item	Mean teachers	Mean students	*t*	*p*
The spaces devoted to teaching do not facilitate the use of active methodologies.	3.53	2.47	5.643	0.000
The infrastructures and equipment are designed for lectures.	3.41	2.79	3.227	0.003
The high number of students per class makes it difficult to use active methodologies.	3.72	3.00	3.505	0.001

*Context in the university.*

### Differences Between the Perception and Opinion of Professors and Their Students Concerning the Teaching-Learning Process (Modes of Organization, Methodological Focuses, and Evaluation Systems)

In this case, to determine whether there are significant differences between perception (Frequency of use) and opinion (Appropriateness of use) and the nature of these differences concerning Modes of Organization (Dimension 1), Methodological Focuses (Dimension 2), and Evaluation Systems (Dimension 3) among the 32 professors and their respective classes, we asked both groups to evaluate what both frequency of use (perception) and appropriateness of use should be (opinion).

As to modes of organization, Table 4 shows that significant differences were obtained in only 3 of the 8 modes presented.

**TABLE 4:** T-Student for related samples. Frequency of use (perception).

Item	Mean teachers	Mean students	*t*	*P*
Seminars	2.53	2.15	1.792	0.015
Practical classes	3.72	2.88	4.754	≤ 0.000
Tutorials	3.34	2.81	2.572	0.083

*Modes of organization.*

As to use of seminars, both professors and their classes believe that this method is used infrequently in the teaching-learning process, but the class believes that it is used even less frequently than do the professors.

As to the use of practical classes and tutorials as a mode of organization, the students’ perception shows that these modes are used less than does their professors’ perception, whereas the professors state that they use these modes some or quite a lot in organizing instruction.

As to opinion concerning appropriate use of the modes of organization presented, Table 5 shows that the professors succeeded in using these modes to a greater extent. The professors also believed more strongly than their students that they used the following modes of teaching organization well: seminars, practical classes, tutorials, independent study and work, and group study and work as organizational modes of instruction.

**TABLE 5:** T-Student for related samples.

Item	Mean teachers	Mean students	T	P
Seminars	3.44	2.82	3.524	0.001
Practical classes	3.78	3.38	2.696	0.011
Tutorials	3.69	3.19	2.853	0.008
Individual study and work	3.53	3.02	2.852	0.008
Group study and work	3.34	2.93	2.163	0.038

*Appropriateness of use (Opinion). Modes of organization.*

It is striking that the opinions of both professors and students agree in assigning similar means to the use of practical classes, tutorials, and independent study and work. Both believe that the use of these modes is appropriate or very appropriate for organizing undergraduate teaching. The opinions of the professors and their students differ, in contrast, on the use of the seminars and group study and work; the professors believe use of these modes to be more appropriate.

For frequency of use of the different methodological focuses, we see statistically significant differences in only 2 of the 11 active methods proposed (Table 6).

**TABLE 6:** T-Student for related samples.

Item	Mean teachers	Mean students	*t*	*p*
Oral presentation of student projects	2.81	2.42	2.065	0.047
Case studies	3.38	2.27	5.245	0.000

*Frequency of use (perception). Methodological focuses.*

These results show that, although professors and students agree in believing that oral presentation of student projects is used little or some, professors believe that it is used more. The use of case studies shows some contradiction; professors believe that they use case studies some and/or a lot in their teaching, whereas their classes perceive that they use case studies less.

As to opinion on the teaching methods, differences arise between professors and class sections on 4 of the 11 focuses. In all four, the professors rate these methods higher, as appropriate or very appropriate for instruction in undergraduate teaching, whereas the students on average score these methods between not very appropriate and appropriate (Table 7).

**TABLE 7:** T-Student.

Item	Mean teachers	Mean students	*t*	*p*
Oral presentation of student projects	3.44	2.81	3.528	0.001
Seminars	3.28	2.80	2.520	0.017
Case studies	3.38	2.88	2.308	0.028
Independent work	3.47	3.06	2.381	0.024

*Opinion (Appropriateness of use) professors/class sections. Methodological focuses.*

Only in the use of independent work can we conclude agreement, as both professors and students believe that independent work is appropriate or very appropriate, although the professors give it a higher score.

For evaluation systems, we observe statistically significant differences in the perception of frequency of use of short-answer tests and oral examinations. In this case, the professors again score them higher (Table 8).

**TABLE 8:** T-Student for related samples.

Item	Mean teachers	Mean students	*t*	*P*
Short-answer tests	2.94	2.34	2.753	0.010
Oral exams	2.72	2.19	2.256	0.031

*Frequency of use (perception). Evaluation systems.*

Although the differences between professors’ opinions and those of their students are noticeable – the use of both tests is between “a little” and “some” – the professors again perceive that they use these systems some, while the students perceive that they use them little (Table 9).

**TABLE 9:** T-Student for related samples.

Item	Mean teachers	Mean students	*t*	*p*
Oral exams	3.31	2.60	3.392	0.002
Papers and projects	3.53	3.20	1.997	0.055
Reports/Memoranda on practical sessions	3.22	2.81	1.982	0.056
Portfolio	2.88	2.35	2.227	0.033

*Appropriateness of use (opinion). Evaluation systems.*

Finally, as to opinion on the use of evaluation systems, we find significant differences on 4 of the 11 evaluation methods presented. These are oral exams, papers and projects, reports and memoranda on practical classes, and the portfolio. In all cases, the professors believe that these methods are more appropriate in instruction, as they score them higher. We would highlight, however, that, despite significant differences of opinion between the professors and their classes on use of papers and projects, professors’ and students’ opinions are closer on this evaluation method; both consider its use as an appropriate or very appropriate method for evaluating students’ learning.

## Conclusion

The study reflects the opinion and perception of both teachers and students on the use of active methodologies. These results can help the university community to improve its teaching practice. It provides knowledge about the different perception that teachers and students have of teaching & learning processes. It is not frequent to have academic studies in which teachers’ and students’ perspectives are part of the same research.

The results shows that the professors believe they are making progress toward a learning-centered model, as the instructors believe that implementation of active methodologies implies new functions in their teaching practice.

Students’ perception of the utility of the methodological focuses are positive. The students generally had a positive attitude toward active learning, especially when they were made aware of the reason for the use of the active techniques. As to use of active methodologies, the findings stress that they foster interdisciplinarity and research and promote the development of learning tools, as well as group work and learning among the students. The main difficulty in implementing these methods is the high number of students per class, which does not make it easy to the develop active methodology. From this study, some recommendations can be made to bring, both to the classroom and university.

### Differences Between the Professor’s Opinion and That of His/Her Students on Active Methodologies

The findings show statistically significant differences in the responses given by the 32 professors and their students on active methodologies, confirming Hypothesis 1.

The content of these results shows that the professors believe they are making progress toward a learning-centered model, as the instructors believe that implementation of active methodologies implies new functions in their teaching practice (Zabalza, 2006, 2011), use of evaluation systems different from those habitually used (Cebrián-de-la-Serna et al., 2015), and obligation to use methods adapted to the characteristics of the students, as well as combined use of lecture and other, active modes of teaching (Salaburu et al., 2011).

This conclusion is also stressed in the study by Yuretich (2003) of students’ perception of the utility of the methodological focuses, which obtained equally positive evaluations for use of lecture and active learning methodologies. Other studies found that the students generally had a positive attitude toward active learning, especially when they were made aware of the reason for the use of the active techniques (Qualters, 2001; Jungst et al., 2003).

As to use of active methodologies, the findings stress that they foster interdisciplinarity and research and promote the development of learning tools, as well as group work and learning among the students.

Ventosa (2004) obtains similar results, highlighting that use of active methods promotes students’ analysis and reflection, contributing to students playing an active role in the acquisition of knowledge. The main difficulty in implementing these methods is the high number of students per class, which does not make it easy to the develop active methodologies (Yuretich, 2003; Machemer and Crawford, 2007; Vreven and McFadden, 2007).

The spaces, infrastructure, and equipment are also considered as designed for imparting lectures, in line with studies by De La Sablonnière et al. (2009).

### Differences Between the Opinion and Perception of Professors and Their Students Concerning the Teaching Process

As to modes of organization, we can conclude that statistically significant differences exist in the use of practical classes and tutorials, which show that they are used more often by the professors than the students indicate. In the opinion of both professors and students, the mode of organization least used is the seminar.

Both professors and students agree in believing that practical classes, tutorials, and independent study and work are the most appropriate modes of organization for instruction. The students view seminars and group study and work as less appropriate (Johnson et al., 1991; Phipps et al., 2001; Vreven and McFadden, 2007; Cavanagh, 2011; Herrmann, 2013).

As to methodological focuses, both professors and students believe that oral presentations of student projects are used little in university classrooms (Carr et al., 2015). The professors believe that they habitually use case studies, although their students perceive them as using this method less.

Professors and students believe that independent work is the most appropriate teaching method. The students state that use of oral presentations of their projects, seminars, and case studies are not very appropriate for instruction, whereas the professors find these methodological focuses to be appropriate (Armbruster et al., 2009; Bruehl et al., 2014).

Finally, we draw the following conclusions concerning the use of evaluation systems. Both professors and students perceive that short-answer tests and oral examinations are not used frequently to evaluate their learning.

In contrast to their students, the professors believe it less appropriate to use both oral examinations and reports and memoranda of practical classes in evaluating students’ learning, perhaps due to the stage fright involved in oral exams and the excessive time required to prepare reports and memoranda (Ituma, 2011).

Professors and students agree in viewing papers and projects as appropriate for evaluating learning, as opposed to the portfolio, which they view as not very appropriate. Some studies, however, consider the portfolio as beneficial as a process for self-regulating learning that requires greater responsibility and motivation (Zimmerman, 2002).

## Limitations and Strengths

The notable strengths of this work are the sample size and the theme, which can contribute. However, despite the novelty and interest of the topic and the results provided in this study. The sample is composed of university students from a single autonomous region and, in addition, no probabilistic sample design was carried out, so the results cannot be generalized.

Further studies should be performed in which other research designs are proposed, On the other hand, it would also be convenient to perform longitudinal researches, with various data collections, in which the effectiveness of use of active methodologies.

The study provides interesting results for the university environment. The study reflects the opinion and perception of both teachers and students on the use of active methodologies. These results can help the university community to improve its teaching practice.

## Data Availability Statement

Publicly available datasets were analyzed in this study. This data can be found here: https://hera.ugr.es/tesisugr/21224043.pdf.

## Ethics Statement

Ethical review and approval was not required for the study on human participants in accordance with the local legislation and institutional requirements. Written informed consent for participation was not required for this study in accordance with the national legislation and the institutional requirements.

## Author Contributions

EC-M: term, conceptualization, methodology, investigation, resources, writing – original draft, writing – review and editing, visualization, and project administration. MR-L: term, conceptualization, methodology, investigation, writing – original draft, writing – review and editing, visualization, and supervision. MC-C: term, conceptualization, methodology, investigation, formal analysis, data curation, writing – original draft, writing – review and editing, visualization, and supervision. All authors contributed to the article and approved the submitted version.

## Conflict of Interest

The authors declare that the research was conducted in the absence of any commercial or financial relationships that could be construed as a potential conflict of interest.

## References

[B1] Alonso F. Méndez R. M. (1999). Modelos de enseñanza de los profesores y enfoques de aprendizaje de los estudiantes: un estudio sobre su relación en la Universidad de Santiago de Compostela. *Adaxe* 14 131–147.

[B2] Armbruster P. Patel M. Johnson E. Weiss M. (2009). Active learning and student-centered pedagogy improve student attitudes and performance in introductory biology. *Education* 8 203–213. 10.1187/cbe.09-03-0025 19723815 PMC2736024

[B3] Attard A. Di Iorio E. Geven K. Santa R. (2010). *Student Centered Leaning. Ghid Pentru Studenţi, Cadre didactice şi Instituţii de Învăţământ Superior (Student Centered Leaning. Guide for Students, Teachers and Higher Education Institutions).* Retreived on www.anosr.ro/wp-content/./07/2012-Toolkit-ICS-cadre-didactice1.pdf (accessed February 29, 2016)

[B4] Barkley E. F. Cross K. P. Major C. H. (2007). *Técnicas de Aprendizaje Colaborativo.* Madrid: Morata.

[B5] Biggs J. (2005). *Calidad del Aprendizaje Universitario.* Madrid: Narcea.

[B6] Bista K. (2011). How to create a learning-centered ESL program. *Engl. Spec. Purposes World* 31 1–13. 10.5430/wjel

[B7] Bonwell C. C. Eison J. A. (1991). *Active Learning: Creating Excitement in the Classroom. ASHE-ERIC Higher Education Report.* Washington DC: George Washington University.

[B8] Boyer E. (1990). *Scholarship Reconsidered: Priorities of the Professoriate. Special Report for the Carnegie Foundation for the Advancement of Teaching.* Atlanta: Jossey Bass.

[B9] Brackin M. (2012). Two-year college faculty and administrator thoughts about the transition to a learning-centered college. *Community Coll. J. Res. Pract.* 36 179–190. 10.1080/10668920802708561

[B10] Bruehl M. Pan D. Ferrer-Vinent J. (2014). Desmystifying the chemistry literature: building information literacy in first-year chemistry students trough student-centered learning and experiment design. *J. Chem. Educ.* 92 52–57. 10.1021/ed500412z

[B11] Buendía L. Colas P. (1997). *Métodos de Investigación en Psicopedagogía.* Madrid: MC Graw-Hill.

[B12] Cabral M. E. Duarte A. M. (2019). La percepción docente sobre su formación en las metodologías activas en el uso de las TIC para el desarrollo de la competencia digital docente de la Carrera de Ciencias de la Educación del Instituto Nacional de Educación Superior. *Rev. Cient. Estud. Investig.* 8 61–62.

[B13] Campbell C. (2012). Learning-centered grading practices. *Leadership* 41 30–33.

[B14] Carr R. Palmer S. Hagel P. (2015). Active learning: the importance of developing a comprehensive measure. *Act. Learn. High. Educ.* 16 173–186. 10.1177/1469787415589529

[B15] Caurcel M. J. Rodríguez A. Romero M. A. (2009). ¿Qué opinan los alumnos universitarios sobre las nuevas metodologías activas de enseñanza? *Prof. Rev. Curriculum Form. Prof.* 13 305–319.

[B16] Cavanagh R. F. (2011). “Establishing the Validity of Rating Scale Instrumentation in Learning Environment Investigations,” in *Applications of Rasch Measurement in Learning Environments Research*, eds Cavanagh R. F. Waugh R. F. (Rotterdam: Sense Publishers), 77–100.

[B17] Cebrián-de-la-Serna M. Bartolomé-Pina A. Cebrián-Robles D. Ruiz-Torres M. (2015). Estudio de los Portafolios en el Practicum: análisis de un PLE-Portafolios. *Relieve* 21 1–19. 10.7203/relieve.21.2.7479

[B18] Chen J. Zhou J. Sun L. Wu Q. Lu H. Tian J. (2015). A new approach for laboratory exercise of pathophysiology in China based on student-centered learning. *Adv. Physiol. Educ.* 39 116–119. 10.1152/advan.00143.2014 26031728

[B19] Chi M. T. H. (2009). Active-Constructive-Interactive: a conceptual framework for differentiating learning activities. *Top. Cogn. Sci.* 1 73–105. 10.1111/j.1756-8765.2008.01005.x 25164801

[B20] Chickering A. W. Gamson Z. F. (1987). Seven principles for good practice in undergraduate education. *AAHE Bull.* 39 3–6. 10.1016/j.iheduc.2004.06.003

[B21] Crisol E. (2013). Opinión y percepción del profesorado y de los estudiantes sobre el uso de las metodologías activas en la Universidad de Granada. Tesis Doctoral Universidad de Granada.

[B22] De La Sablonnière R. Taylor D. M. Sadykova N. (2009). Challenges of applying a student-centred approach to learning in the context of Education in Kyrgystan. *Int. J. Educ. Dev.* 29 628–634.

[B23] De Miguel M. (2006). *Propuestas para la Renovación de las Metodologías Educativas en la Universidad.* Madrid: Secretaría General Técnica del MEC.

[B24] De Miguel M., and coord. (2009). *Metodologías de Enseñanza y Aprendizaje para el Desarrollo de Competencias: Orientaciones para el Profesorado Universitario ante el Espacio Europeo de Educación Superior.* Madrid: Alianza.

[B25] Dominguez A. Truyol M. E. Zavala G. (2019). Professional development program to promote active learning in an engineering classroom. *IJEE* 35 424–433.

[B26] Exley K. Dennick R. (2007). *Enseñanza en pequeños grupos en Educación Superior.* Madrid: Narcea.

[B27] Felder R. M. Brent R. (1996). Navigating the bumpy road to student-centered instruction. *Coll. Teach.* 44 43–47. 10.1080/87567555.1996.993.3425

[B28] Fernández A. (2009). Metodologías activas para la formación en competencias. *Educ. Siglo XXI* 24 35–56.

[B29] Fernández-Pérez M. (1989). *Así Enseña Nuestra Universidad.* Salamanca: Hispagraphis.

[B30] Fink L. D. (2003). *Creating Significant Learning Experiences: An Integrated Approach to Designing College Courses.* San Francisco, CA: Jossey-Bass.

[B31] García Valcárcel A. (1993). Análisis de los modelos de enseñanza empleados en el ámbito universitario. *Rev. Esp. Pedag.* 194 27–53.

[B32] Gargallo-López B. Pérez-Pérez C. Verde-Peleato I. García-Félix E. (2017). Estilos de aprendizaje en estudiantes universitarios y enseñanza centrada en el aprendizaje. *Relieve* 23:46 10.7203/relieve.23.2.9078

[B33] Gibbs G. (1994). *Learning in Teams: A Student’s Guide.* Oxford: Oxford Brookes University.

[B34] Gómez P. Gil A. J. (2018). El estilo de aprendizaje y su relación con la educación entre pares. *Rev. Investig. Educ.* 36 221–237. 10.6018/rie.36.1.233731

[B35] Hannafin M. (2012). “Student-Centered Learning,” in *Encyclopedia of the Sciences of Learning*, ed. Seel N. M. (Nueva York, NY: Springer), 3211–3214.

[B36] Heise B. Himes D. (2010). The course council: an example of student-centered learning’ educational innovation. *Release* 29 1–3. 10.3928/01484834-20100115-04 20143757

[B37] Hernández R. (2012). Does continuous assessment in higher education support student learning? *High. Educ.* 64 489–502. 10.1007/s10734-012-9506-7

[B38] Hernández R. Fernández C. Baptista P. (2006). *Metodología de Investigación.* Mexico: McGraHill.

[B39] Herrmann K. J. (2013). The impact of cooperative learning on student engagement: results from an intervention. *Act. Learn. High. Educ.* 14 175–187. 10.1177/1469787413498035

[B40] Hunt L. Chalmers D. (2013). *University Teaching in Focus: A Learning-Centred Approach.* Abingdon: Routledge.

[B41] Hynes M. (2017). Students-as-producers: developing valuable student-centered research and learning opportunities. *Int. J. Res. Stud. Educ.* 7 1–13. 10.5861/ijrse.2017.1858

[B42] Imbernón F. Medina J. L. (2006). “Metodología participativa en el aula universitaria. La participación del alumnado,” in *Propuestas para el Cambio Docente en la Universidad*, eds Martínez M. Carrasco S. (Barcelona: Octaedro), 91–122.

[B43] Imbernón F. Medina J. L. (2008). *Metodología Participativa en Aula Universitaria. La Participación del Alumnado.* Barcelona: Octaedro.

[B44] Ituma A. (2011). An evaluation of students’ perceptions and engagement with e-learning components in a campus based university. *Act. Learn. High. Educ.* 12 57–68. 10.1177/1469787410387722

[B45] Johnson D. W. Johnson R. Smith K. (1991). *Active Learning: Cooperation in the College Classroom.* Edina, MN: Interaction Book Company.

[B46] Johnson D. W. Johnson R. T. Holubec E. J. (1999). *El Aprendizaje Cooperativo en el Aula.* Madrid: Paidós.

[B47] Jungst S. E. Thompson J. R. Atchison G. J. (2003). Academic controversy: fostering constructive conflict in the classroom. *J. Nat. Resour. Life Sci. Educ.* 32 36–42.

[B48] Kember D. (2009). Promoting student-centred forms of learning across an entire university. *High. Educ.* 58 1–13. 10.1007/s10734-008-9177-6

[B49] Kember D. Kwan K. P. (2000). Lecturers’ approaches to teaching and their relationship to conceptions of good teaching. *Inst. Sci.* 28 469–490.

[B50] Kolb D. (1976). *The Learning Style Inventory: Technical Manual.* Boston, MA: McBer and Company.

[B51] Koles P. Nelson S. Stolfi A. Parmelee D. DeStephen D. (2005). Active learning in a year 2 pathology curriculum. *Med. Educ.* 39 1045–1055. 10.1111/j.1365-2929.2005.02248.x 16178832

[B52] Lammers W. J. Murphy J. J. (2002). A profile of teaching techniques used in the university classroom: a descriptive profile of a US public university. Act. Learn. *High. Educ.* 3 54–67. 10.1177/1469787402003001005

[B53] Learreta B. Montil M. González-Álvarez A. Asensio A. (2009). Percepción del alumnado ante el uso de metodologías activas de enseñanza como respuesta a las demandas del espacio europeo de educación superior: un estudio de caso. *Apunts Educ. Fís. Deportes.* 1 92–98.

[B54] León M. J. Crisol E. (2011). Diseño de cuestionarios (OPPUMAUGR Y OPEUMAUGR): La opinión y la percepción del profesorado y de los estudiantes sobre el uso de las metodologías activas en la universidad. *Profesorado*, 15, 271–298.

[B55] López-Noguero F. (2007). *Metodología Participativa en la Enseñanza Universitaria.* Madrid: Narcea.

[B56] López-Pastor V. M. Castejón J. Sicilia-Camacho A. Navarro-Adelantado V. Webb G. (2011). The process of creating a cross–university network for formative and shared assessment in higher education in Spain and its potential applications. *Innov. Educ. Teach. Int.* 48 79–90. 10.1080/14703297.2010.543768

[B57] Lucieer S. M. Van der Geest J. N. ElóiSantos S. M. Delbone de Faria R. M. Jonker L. Visscher C. H. (2016). The development of self-regulated learning during the pre-clinical stage of medical school: a comparison between a lecturebased and a problem-based curriculum. *Adv. Health Sci. Educ.* 21 93–104. 10.1007/s10459-015-9613-1 26018998 PMC4749637

[B58] Machemer P. L. Crawford P. (2007). Student perceptions of active learning in a large cross-disciplinary classroom. *Act. Learn. High. Educ.* 8 9–30. 10.1177/1469787407074008

[B59] Maclellan E. (2008). The significance of motivation in student-centred learning: a reflective case study. *Teach. High. Educ.* 13 411–421.

[B60] Marín M. Teruel M. P. (2004). La formación del docente universitario: necesidades y demandas desde el alumnado. *Rev. Interuniv. Form. Prof.* 18 137–151. 10.3109/0142159X.2011.579199 21696272

[B61] McLean M. Gibbs T. (2010). Twelve tips to designing and implementing a learner-centered curriculum: prevention is better than cure. *Med. Teach.* 32 225–230. 10.3109/01421591003621663 20218837

[B62] Menachery E. P. Wright S. M. Howell E. E. Knight A. M. (2008). Physicianteacher characteristics associated with learner-centered teaching skills. *Med. Teach.* 30 137–144. 10.1080/01421590801942094 18576184

[B63] Meyers C. Jones T. B. (1993). *Promoting Active Learning: Strategies for the College Classroom.* San Francisco, CA: Jossey-Bass Inc.

[B64] Ministry of Education and Culture [MEC] (2006). *Propuestas para la Renovación de las Metodologías Educativas en la Universidad.* Madrid: Secretaría General Técnica del MEC.

[B65] Monereo C. Pozo J. I. (2006). *La Universidad ante la Nueva Cultura Académica. Enseñar y Aprender Para la Autonomía.* Madrid: Síntesis.

[B66] Mostrom A. Blumberg P. (2012). Does learning-centered teaching promote grade improvement? *Innov. High. Educ.* 37 397–405. 10.1007/s10755-012-9216-1

[B67] Moust J. Bouhuijs P. Schmidt H. (2007). *El Aprendizaje Basado en Problemas: Guía del Estudiante.* Cuenca: Ediciones de la Universidad de Castilla-La Mancha.

[B68] Nitza D. (2013). Learning-centered teaching and backward course design-From transferring knowledge to teaching skills. *J. Int. Res.* 9 329–338. 10.19030/jier.v9i4.8084

[B69] Phipps M. Phipps C. Kask S. Higgins S. (2001). University students’ perceptions of cooperative learning: implications for administrators and instructors. *J. Exp. Educ.* 24 14–21. 10.1177/105382590102400105

[B70] Prieto L., and coord. (2008). *La Enseñanza Universitaria Centrada en el Aprendizaje.* Barcelona: Octaedro.

[B71] Prince M. (2004). Does active learning work? A review of the research. *J. Eng. Educ.* 93 223–232. 10.1002/j.2168-9830.2004.tb00809.x

[B72] Qualters D. M. (2001). *Using classroom assessment data to improve student learning Classroom Assessment Guidebook.* Boston, MA: Northeastern University.

[B73] Roy E. B. McMahon G. T. (2012). Videobased cases disrupt Deep critical thinking in problem-based learning. *Med. Educ.* 46 426–435. 22429179 10.1111/j.1365-2923.2011.04197.x

[B74] Rué J. (2007). *Enseñar en la Universidad.* Madrid: Narcea.

[B75] Salaburu P. Haug G. Mora J. G. (2011). *España y el Proceso de Bolonia. Un Encuentro Imprescindible.* Madrid: Academia Europea de Ciencias y Artes.

[B76] Samuelowicz K. Bain J. D. (2002). Identifying academics’ orientatios to assessment practice. *High. Educ.* 43 173–201. 10.1023/A:1013796916022

[B77] Sánchez M. (2008). *Como Enseñar en las Aulas Universitarias a Través del Estudio de Casos.* Zaragoza: Universidad de Zaragoza.

[B78] Schweisfurth M. (2015). Learner-centred pedagogy: towards a post-2015 agenda for teaching and learning. *Int. J. Educ. Dev.* 40 259–266.

[B79] Sierra Bravo R. (1988). *Técnicas de INVESTIGACIÓN SOCIal. Teoría y Ejercicios.* Madrid: Paraninfo.

[B80] Sue T. (2014). Student-centred learning: a humanist perspective. *Teach. High. Educ.* 19 266–275.

[B81] Tagg J. (2003). *The Learning Paradigm College.* Bolton, MA: Anker Publishing Company, Inc.

[B82] Tessier J. (2007). Small-group peer teaching in an introductory biology classroom. *J. Coll. Sci. Teach.* 36 64–69.

[B83] Tien L. T. Roth V. Kampmeier J. A. (2002). Implementation of a peer-led team learning instructional approach in an undergraduate organic chemistry course. *J. Res. Sci. Teach.* 39 606–632. 10.1002/tea.10038

[B84] Vallejo-Ruiz M. Molina-Saorín J. (2011). Análisis de las metodologías activas en el grado de maestro en educación infantil: la perspectiva del alumnado. *REIFOP* 14 207–217.

[B85] Ventosa V. J. (2004). *Métodos Activos y Técnicas de Participación para Educadores y Formadores.* Madrid: Editorial CCS.

[B86] Vreven D. McFadden S. (2007). An empirical assessment of cooperative groups in large, time-compressed, introductory courses. *Innov. High. Educ.* 32 85–92. 10.1007/s10755-007-9040-1

[B87] Yuretich R. F. (2003). Encouraging critical thinking: measuring skills in large introductory science classes. *J. Coll. Sci. Teach.* 33 40–45.

[B88] Zabalza M. A. (2006). “El practicum y la formación del profesorado: balance y propuesta para las nuevas titulaciones,” in *La Formación del Profesorado y la Mejora de la Educación*, eds Escudero J. M. Gómez A. L. (Barcelona: Octaedro), 311–334.

[B89] Zabalza M. A. (2011). Metodología docente. El espacio europeo de educación superior. ¿Hacia dónde va la Universidad Europea?. *Rev. Doc. Univ.* 9 75–98.

[B90] Zamora-Polo F. Sánchez-Martín J. (2019). Teaching for a Better World. Sustainability and Sustainable Development Goals in the Construction of a Change-Maker University. *Sustainability* 11:4224 10.3390/su11154224

[B91] Zimmerman B. J. (2002). Becoming a self-regulated learner: an overview. *Theory Pract.* 41 65–70. 10.1207/s15430421tip4102_2

